# Hemorrhoid is associated with increased risk of peripheral artery occlusive disease: A nationwide cohort study

**DOI:** 10.1016/j.je.2016.12.015

**Published:** 2017-03-03

**Authors:** Wei-Syun Hu, Cheng-Li Lin

**Affiliations:** a School of Medicine, College of Medicine, China Medical University, Taichung, Taiwan; b Division of Cardiovascular Medicine, Department of Medicine, China Medical University Hospital, Taichung, Taiwan; c Management Office for Health Data, China Medical University Hospital, Taichung, Taiwan

**Keywords:** Cohort study, Comorbidity, Hemorrhoid, Peripheral artery occlusive disease

## Abstract

**Background:**

This study was conducted to evaluate the association between hemorrhoid and risk of incident peripheral artery occlusive disease (PAOD).

**Methods:**

Using the Taiwanese Longitudinal Health Insurance Database 2000, we compared the incident PAOD risk between the hemorrhoid and the non-hemorrhoid cohorts. Both of these cohorts were followed up from the index date until the date of PAOD diagnosis, withdrawal from the National Health Insurance program, or the end of 2011.

**Results:**

The mean follow-up period was 6.82 (standard deviation [SD], 3.22) and 6.70 (SD, 3.23) years in the hemorrhoid and non-hemorrhoid cohorts, respectively. The plot of the Kaplan–Meier analysis showed that, by the end of the 12-year follow-up period, the cumulative incidence of PAOD was significantly higher for the hemorrhoid cohort than for the non-hemorrhoid cohort (log-rank test: *P* < 0.001).

**Conclusions:**

A significantly increased PAOD risk in patients with hemorrhoids was found in this nationwide cohort study.

## Highlights

• This study is a nationwide population-based cohort study.
• This study aimed to evaluate the association between hemorrhoid and risk of PAOD.
• Increased risk of PAOD in patients with hemorrhoids was found in this study.

## Introduction

Hemorrhoid, which varies clinically from asymptomatic to manifestations of bleeding, prolapse, and thrombosis, is becoming a huge medical burden worldwide.^1^, ^2^, ^3^, ^4^ Several theories have been proposed for the development of the hemorrhoid; among them, inflammation is one of the pathogenic processes that has gained attention recently.^1^, ^2^, ^3^, ^4^, ^5^, ^6^

Peripheral artery occlusive disease (PAOD) is one of the leading causes of mortality worldwide.^7^, ^8^, ^9^, ^10^ Patients with PAOD are usually asymptomatic and are easily overlooked.^7^, ^8^, ^9^, ^10^, ^11^, ^12^ The risk factors of developing PAOD have been well established in previous investigations.^13^, ^14^, ^15^

Matrix metalloproteinases (MMPs), key players in the pathogenesis of PAOD, have recently been reported to be associated with hemorrhoid development.^5^, ^16^, ^17^, ^18^ However, no study has addressed the relationship between hemorrhoid and the risk of incident PAOD. Therefore, this study was designed to evaluate the association between hemorrhoid and the subsequent PAOD risk using a nationwide population-based database.

## Methods

### Data source

A nationwide population-based retrospective cohort study was performed using the Taiwanese Longitudinal Health Insurance Database 2000 (LHID2000). The LHID2000 comprises one million randomly sampled beneficiaries enrolled in the National Health Insurance (NHI) program, which collected all records on these individuals from 1996 to 2011. The NHI program includes the complete medical information of more than 23.74 million Taiwanese residents, with a coverage rate of over than 99%.^19^ The NHI program and LHID2000 have been described in detail previously.^20^, ^21^ The identification numbers of patients have been scrambled to protect the privacy of insured residents before releasing the LHID2000. Diseases diagnoses were identified and coded using the International Classification of Diseases, 9th Revision, Clinical Modification (ICD-9-CM). The Ethics Review Board of China Medical University and Hospital in Taiwan approved this study (CMUH-104-REC2-115).

### Sampled participants

Subjects with hemorrhoids (ICD-9-CM code 455) newly diagnosed from January 2000 through December 2010 were included in the hemorrhoid cohort. The first date of hemorrhoid diagnosed was defined as the entry date. We excluded patients with a history of PAOD (ICD-9-CM codes 440.2, 440.3, 440.8, 440.9, 443, 444.22, 444.8, 447.8, and 447.9) before the entry date or those aged <20 years. The non-hemorrhoid cohort was identified from the LHID2000 during the same period of 2000–2010, with exclusion criteria similar to the hemorrhoid cohort. Patients in the hemorrhoid and non-hemorrhoid cohorts were selected by 1:1 frequency matching by sex, age (every 5-year span), index year, and comorbidities of diabetes (ICD-9-CM code 250), hypertension (ICD-9-CM codes 401–405), hyperlipidemia (ICD-9-CM code 272), chronic obstructive pulmonary disease (COPD) (ICD-9-CM codes 491, 492, and 496), heart failure (ICD-9-CM code 428), coronary artery disease (CAD) (ICD-9-CM codes 410–414), stroke (ICD-9-CM codes 430–438), and asthma (ICD-9-CM code 493). The comorbidities diagnosed before the end of the study were included for adjustment. Both these cohorts were followed up from the index date until the date of PAOD diagnosis, withdrawal from the NHI program, or the database ended (December 31, 2011), whichever came first.

### Statistical analysis

Distributions of demographic variables, including sex, age, and comorbidities were compared between the hemorrhoid and the non-hemorrhoid cohorts. The categorical variables were analyzed using the chi-square test, and the continuous variables of the baseline characteristics of these cohorts were analyzed using the Student *t*-test. To assess the difference of the cumulative incidence of PAOD between the hemorrhoid and non-hemorrhoid cohorts, we applied the Kaplan–Meier analysis and the log-rank test. We computed the incidence density rate (per 1000 person-years) of PAOD for each cohort. Cox proportional hazard model was used to assess the risk of PAOD between the hemorrhoid and the non-hemorrhoid cohorts. Sex, age, and comorbidities of diabetes, hypertension, hyperlipidemia, COPD, heart failure, CAD, stroke, and asthma were included in the multivariable model for adjustment. We estimated the hazard ratios (HRs) and 95% confidence intervals (CIs) using the Cox model. We performed all statistical analyses using SAS 9.4 (SAS Institute Inc., Cary, NC, USA), with *P* < 0.05 in two-tailed tests considered significant.

## Results

Eligible study patients included 37,992 patients with hemorrhoids and 37,992 patients without hemorrhoids (Table 1). No significant differences regarding the distributions of sex, age, and comorbidities between the hemorrhoid and non-hemorrhoid cohorts were found. Males represented the majority of the study cohorts (54.3% vs. 54.2%); most people were less than 50-years-old (60.5% vs. 60.5%). The mean age of the patients in the hemorrhoid and the non-hemorrhoid cohorts was 47.2 (standard deviation [SD], 15.8) and 47.0 (SD, 16.2) years, respectively.

**Table 1:** Demographic characteristics and comorbidities in patients with and without hemorrhoids.

Variable	Hemorrhoid		*P* value
	No	Yes	
	*n* = 37,992	*n* = 37,992	
Sex			0.93
Female	17,387 (45.8)	17,375 (45.7)	
Male	20,605 (54.2)	20,617 (54.3)	
Age, years, mean (SD)	47.0 (16.2)	47.2 (15.8)	0.05
Age groups, years			0.89
≤49	22,966 (60.5)	22,983 (60.5)	
50–64	8855 (23.3)	8865 (23.3)	
≥65	6161 (16.2)	6144 (16.2)	
Comorbidity			
Diabetes	4125 (10.9)	4157 (10.9)	0.71
Hypertension	13,400 (35.2)	13,405 (35.3)	0.97
Hyperlipidemia	10,920 (28.7)	10,968 (28.8)	0.70
COPD	3924 (10.3)	3957 (10.4)	0.69
Heart failure	1523 (4.01)	1558 (4.10)	0.52
CAD	7419 (19.5)	7454 (19.6)	0.75
Stroke	7841 (20.6)	7869 (20.7)	0.80
Asthma	2391 (6.29)	2415 (6.36)	0.72

CAD, coronary artery disease; COPD, chronic obstructive pulmonary disease; SD, standard deviation. Values are reported as *n* (%), unless otherwise noted.

**Fig. 1:**
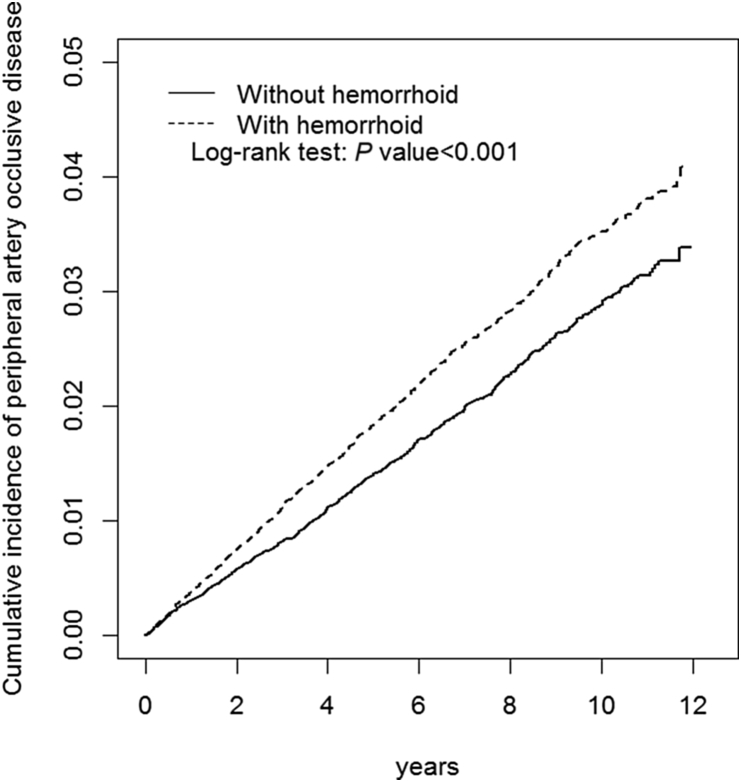
Cumulative incidence curves of peripheral artery occlusive disease (PAOD) for patients with and without hemorrhoid.

The mean follow-up period was 6.82 (SD, 3.22) and 6.70 (SD, 3.23) years in the hemorrhoid and non-hemorrhoid cohorts, respectively. The plot of the Kaplan–Meier analysis showed that, by the end of the 12-year follow-up period, the cumulative incidence of PAOD was significantly higher for the hemorrhoid cohort than for the non-hemorrhoid cohort (log-rank test: *P* < 0.001) (Fig. 1).

The overall, sex-, age-, and comorbidity-specific incidence density rates and HR of these two cohorts are shown in Table 2. The overall incidence rate of PAOD was significantly higher in the hemorrhoid cohort than in the non-hemorrhoid cohort (3.61 vs. 2.88 per 1000 person-years) with an adjusted hazard ratio (aHR) of 1.25 (95% CI, 1.14–1.38). The risk of PAOD for the hemorrhoid relative to the non-hemorrhoid cohort was significantly higher in both women (aHR 1.27; 95% CI, 1.10–1.47) and men (aHR 1.24; 95% CI, 1.08–1.41). The incidence of PAOD increased with age in both cohorts, and the age-specific aHR of PAOD for the hemorrhoid relative to the non-hemorrhoid cohort was significantly higher for those aged 50–64 years (aHR 1.22; 95% CI, 1.03–1.45) and ≧65 years (aHR 1.32; 95% CI, 1.15–1.53). The risk of PAOD for the hemorrhoid relative to non-hemorrhoid cohort was significantly higher for those without comorbidity (aHR 1.62; 95% CI, 1.15–2.27) and with comorbidity (aHR 1.22; 95% CI, 1.11–1.35). The results of the univariable and multivariable Cox proportional hazards regression models for analyzing the risk of variables contributing to PAOD are shown in Table 3. The aHR of PAOD increased 1.04-fold (95% CI, 1.04–1.05) with age (per year). The risk of PAOD was greater in patients with comorbidities, namely diabetes (aHR 1.40; 95% CI, 1.25–1.57), hypertension (aHR 1.73; 95% CI, 1.51–1.98), hyperlipidemia (aHR 1.12; 95% CI, 1.01–1.25), and CAD (aHR 1.46; 95% CI, 1.30–1.63).

**Table 2:** Comparison of incidence and hazard ratio of peripheral artery occlusive disease stratified by sex, age, and comorbidity between patients with and without hemorrhoids.

Variable	Without hemorrhoid					With hemorrhoid				
	Event	PY	Rate^a^	Crude HR (95% CI)	Adjusted HR^b^ (95% CI)	Event	PY	Rate^a^	Crude HR (95% CI)	Adjusted HR^b^ (95% CI)
All	732	254,370	2.88	1.00	1.00	934	259,014	3.61	1.25 (1.14, 1.38)***	1.25 (1.14, 1.38)***
Sex										
Female	333	118,731	2.80	1.00	1.00	420	119,998	3.50	1.25 (1.08, 1.44)**	1.27 (1.10, 1.47)**
Male	399	135,639	2.94	1.00	1.00	514	139,017	3.70	1.26 (1.10, 1.43)***	1.24 (1.08, 1.41)**
*P* for interaction									0.94	
Age group, years										
≤49	177	162,043	1.09	1.00	1.00	213	165,557	1.29	1.18 (0.96, 1.44)	1.18 (0.96, 1.44)
50–64	241	56,950	4.23	1.00	1.00	294	57,156	5.14	1.22 (1.03, 1.44)*	1.22 (1.03, 1.45)*
≥65	314	35,377	8.88	1.00	1.00	427	36,301	11.8	1.33 (1.15, 1.53)***	1.32 (1.15, 1.53)***
*P* for interaction									0.95	
Comorbidity^c^										
No	53	107,527	0.49	1.00	1.00	87	110,243	0.79	1.60 (1.14, 2.25)**	1.62 (1.15, 2.27)**
Yes	679	146,843	4.62	1.00	1.00	847	148,771	5.69	1.23 (1.11, 1.36)***	1.22 (1.11, 1.35)***
*P* for interaction									0.15	

CI, confidence interval; HR, hazard ratio; PY, person-years.

*P < 0.05, **P < 0.01, ***P < 0.001.

a Incidence rate, per 1000 person-years.

b Adjusted for age, sex, and comorbidities of diabetes, hypertension, hyperlipidemia, chronic obstructive pulmonary disease, heart failure, coronary artery disease, stroke, and asthma.

c Patients with diabetes, hypertension, hyperlipidemia, chronic obstructive pulmonary disease, heart failure, coronary artery disease, stroke, and asthma were classified as the comorbidity group.

**Table 3:** Hazard ratios of peripheral artery occlusive disease in association with sex, age, and comorbidities in univariable and multivariable Cox regression models.

Variable	Crude		Adjusted^a^	
	HR	(95% CI)	HR	(95% CI)
Hemorrhoid	1.25	(1.14, 1.38)***	1.25	(1.14, 1.38)***
Sex (Men vs Women)	1.05	(0.96, 1.16)	0.91	(0.83, 1.00)
Age, years	1.06	(1.05, 1.06)***	1.04	(1.04, 1.05)*
Comorbidities (yes vs no)				
Diabetes	2.90	(2.60, 3.24)***	1.40	(1.25, 1.57)***
Hypertension	4.95	(4.44, 5.51)***	1.73	(1.51,1.98)***
Hyperlipidemia	2.37	(2.15, 2.61)***	1.12	(1.01, 1.25)*
COPD	2.51	(2.27, 2.79)***	0.99	(0.88, 1.12)
Heart failure	3.47	(2.99, 4.02)***	0.98	(0.84, 1.15)
CAD	4.08	(3.70, 4.49)***	1.46	(1.30, 1.63)***
Stroke	3.15	(2.86, 3.47)***	1.10	(0.99, 1.23)
Asthma	1.79	(1.57, 2.03)***	0.97	(0.85,1.12)

CI, confidence interval; CAD, coronary artery disease; COPD, chronic obstructive pulmonary disease; HR, hazard ratio.

*P < 0.05, **P < 0.01, ***P < 0.001.

a Multivariable analysis, including age, sex, and comorbidities of diabetes, hypertension, hyperlipidemia, COPD, heart failure, CAD, stroke, and asthma.

## Discussion

To the best of our knowledge, this study is the first to identify the association between hemorrhoid and risk of incident PAOD. After adjustment for age, gender, and comorbidities, patients with hemorrhoids had a significantly increased risk of developing subsequent PAOD.

The strength of our investigation is that it is based upon a nationwide population dataset with an adequate number of participants who were followed-up for a very long time to enable significant analysis.^19^ Therefore, the association between hemorrhoid and the subsequent PAOD risk was highly convincing.

In this study, men represented the majority of the hemorrhoid patients (54.3%), and the mean age of the patients with hemorrhoids was 47.2 years. Our findings are comparable with those of previous investigations,^1^, ^2^, ^3^, ^4^ further verifying the reliability of National Health Insurance Research Database hemorrhoid cohort data. In this study, we found that hemorrhoid patients had a 25% increased risk of subsequent PAOD development after adjustment for age, gender, and other medical comorbidities. Additionally, the risk of developing PAOD for the hemorrhoid cohort relative to the non-hemorrhoid cohort was significantly higher in the subgroups of older patients and those with no comorbidity, further implying that the association between hemorrhoid and risk of incident PAOD might be unrelated to underlying comorbidities. More studies are mandatory to substantiate our findings.

A subgroup analysis was conducted to evaluate the impact of hemorrhoid and respective medical comorbidity on the development of PAOD. Our finding is compatible with the current knowledge that the risk of developing PAOD is higher among patients with diabetes, hypertension, and CAD.^13^, ^14^, ^15^ Though the impact of hemorrhoid on the development of PAOD was not as high as that for conventional PAOD-associated risk factors,^13^, ^14^, ^15^ hemorrhoid still conferred a significantly increased risk of PAOD, with steadily increased during the 12-year follow-up period, after minimizing confounding factors. Further large-scale studies to explore the association between hemorrhoid and subsequent PAOD risk are worthwhile.

Several possible factors may explain the higher PAOD risk among patients with hemorrhoids.^1^, ^2^, ^3^, ^4^, ^22^, ^23^, ^24^ First, the role of inflammation, which has been well established as a major trigger of acute atherosclerotic events,^25^, ^26^, ^27^ in the development of hemorrhoid has recently gained increasing attention.^5^, ^6^ Second, patients with hemorrhoids tend to lead a sedentary lifestyle and be obese, factors which are strongly associated with the development of PAOD.^1^, ^2^, ^3^, ^4^, ^22^, ^23^, ^24^ Further investigations are warranted to verify the role of PAOD in hemorrhoid and to explore the underlying mechanism.

### Limitations

First, the diagnoses of diseases were identified and coded using the ICD-9-CM, and the severity and classification of hemorrhoid, PAOD, and other medical comorbidities could not be obtained via the LHID2000. Second, we could not retrieve detailed information regarding family history of PAOD, smoking, obesity, and physical activity from the LHID2000. Finally, this study is a retrospective cohort study, with certain inherent methodological limitations.

## Conclusions

In conclusion, a significantly increased PAOD risk in patients with hemorrhoids was found in this nationwide cohort study. Further studies are required to confirm the clinical significance of our findings and to explore the underlying mechanism.

## Conflicts of interest

None declared.
